# Dr. Chas. L. Stiles

**Published:** 1916-07

**Authors:** 


**Dr. Chas. L. Stiles,** Geneva 1865, died April 22. He had been a practitioner of Owego since his graduation and was prominent in medical organizations being an honorary member of the Elmira Academy of Medicine.

